# Patient-reported function, quality of life and prosthesis wear in adults born with one hand: a national cohort study

**DOI:** 10.1177/17531934231222017

**Published:** 2023-12-21

**Authors:** Ida Neergård Sletten, Kari Klungsøyr, Andrew Garratt, Jarkko Jokihaara

**Affiliations:** 1 Division of Orthopaedic Surgery, Oslo University Hospital, Oslo, Norway; 2 Division of Mental and Physical Health, Norwegian Institute of Public Health, Bergen, Norway; 3 Department of Global Public Health and Primary Care, University of Bergen, Bergen, Norway; 4 Division for Health Services, Norwegian Institute of Public Health, Oslo, Norway; 5 Department of Hand Surgery, Tampere University Hospital, Tampere, Finland; 6 Faculty of Medicine and Health Technology, Tampere University, Tampere, Finland

**Keywords:** Congenital anomaly, limb reduction defect, transverse deficiency upper limb, upper limb function, upper limb prosthesis

## Abstract

We invited individuals aged above 16 years with a congenital transverse reduction deficiency at and above the wrist born in Norway between 1970 and 2006 to complete the short version of the Disabilities of the Arm, Shoulder and Hand Outcome Measure, the 5-Level EuroQoL-5-Dimension instrument, the RAND 36-Item Short Form Health Survey and a single-item questionnaire on arm function, appearance, pain and prosthesis wear. Of 154 eligible participants, 58 (38%) responded. Their scores were not different from the general population. All had been offered prostheses, and 56 (97%) had been fitted at a median age of 1 year (interquartile range 0–2.8). Of the participants, 37 (64%) were still prosthesis wearers, while 21 (36%) were non-wearers or using gripping devices only. Prosthesis wearers had higher levels of ‘vitality’ as assessed by the RAND-36 and rated their arm appearance higher, but there were no other score differences, indicating that prosthesis rejection is not associated with worse functional outcomes.

**Level of evidence:** III

## Introduction

Transverse reduction deficiencies at and above the wrist level (TRDAWs) are rare congenital upper limb anomalies (CULAs) (Ekblom et al., 2010; Koskimies et al., 2011). They are characterized by a short arm without a hand and classified as symbrachydactyly (when nubbins are present) or transverse deficiency (Goldfarb et al., 2020; Kallemeier et al., 2007). The defect is most often unilateral and at the forearm level (Koskimies et al., 2011). Children with TRDAWs have only mild impairment in upper extremity function (James et al., 2006; Shoghi et al., 2022) and a normal health-related quality of life (HRQoL) (James et al., 2006; Johansen et al., 2016a; Shoghi et al., 2022); however, in adults, the association between TDRAW and function, pain or HRQoL is unclear (Dwivedi et al., 2022; Johansen et al., 2016b; Postema et al., 2016).

Norwegian children and adults with TRDAWs have for several decades been offered prostheses free of cost at the point of delivery. Currently, we fit a passive prosthesis at 6 months, an active (myoelectric) prosthesis at 3–4 years and offer activity-specific gripping devices at all ages. This pattern of fitting is similar across Nordic countries but is different from many other parts of the world. Prostheses do not seem to improve children’s physical function or HRQoL (James et al., 2006). However, the rationale for an early start to prosthesis wear is based on the assumption that it provides advantages during childhood that carry into adulthood. Some children reject their prostheses at an early age or during adolescence (Davids et al., 2006; Huizing et al., 2010; Postema et al., 1999; Scotland and Galway, 1983) owing to lack of function and discomfort (Smail et al., 2021), and absence of sensory feedback from the distal extremity. The benefits of prostheses have not been investigated in adults with TRDAWs.

The primary aim of this study in a Norwegian cohort was to investigate disability associated with unilateral TDRAW in adults compared with the general population using patient-reported outcome measures (PROMs) for upper extremity function and other aspects of HRQoL. The secondary aim was to assess any benefit of long-term prosthesis wearing by comparing PROM outcomes in adult wearers and non-wearers.

## Methods

The Regional Committee for Medical and Health Research Ethics and the Data Protection Officer at Oslo University Hospital approved this study. It was conducted according to the Helsinki Declaration, and reporting follows the Strengthening the Reporting of Observational Studies in Epidemiology (STROBE) statement (Vandenbroucke et al., 2007).

With a mean of 53,437 births per year in Norway between 1972 and 2006, and a Nordic incidence of upper limb transverse deficiency of 0.8 per 10,000 newborns (Ekblom et al., 2010; Koskimies et al., 2011), we estimated there would be a study population of 156 persons. We used the national Medical Birth Registry of Norway (MBRN) and the CULA (congenital upper limb anomaly) North Oslo Registry to identify eligible study participants; individuals aged above 16 years with TRDAWs born in Norway between 1970 and 2006. The reporting of pregnancies, births and congenital anomalies to the MBRN is mandatory (Irgens, 2000). Previous classification systems were used until 1999, but the MBRN has re-coded all limb reduction data before the change to the International Classification of Diseases Tenth Revision (ICD-10) so that all data from 1970 is now coded identically according to ICD-10 (Klungsøyr et al., 2019). The CULA North Oslo Registry includes all patients with TRDAWs assessed at Oslo University Hospital starting in 1999. In contrast to the MBRN, a congenital hand surgeon (INS) classified all anomalies at inclusion.

Our study approval allowed us to contact all eligible study participants by mail, including one reminder for non-respondents. Participants signed a written consent at inclusion. The questionnaire asked about the side and level of the TRDAW according to a picture with five zones (shoulder, upper arm, lower arm, upper forearm, lower forearm), other congenital anomalies, any CULAs in relatives and whether the participant had undergone any related surgery.

The participants completed the short version of the Disabilities of the Arm, Shoulder and Hand Outcome Measure (QuickDASH) (Beaton et al., 2005; Hudak et al., 1996), the 5-Level EuroQoL-5-Dimension (EQ-5D-5L) instrument (EuroQolGroup, 1990) and the RAND 36-Item Short Form Health Survey (RAND-36) (Hays and Morales, 2001). Data for the Norwegian general population are available for these PROMs (Aasheim and Finsen, 2014; Garratt et al., 2022; Garratt and Stavem, 2017). There is no Norwegian scoring algorithm for the EQ-5D-5L index, and current national recommendations were followed, including the use of the UK crosswalk value set, which allows values for the EQ-5D-5L to be obtained by means of mapping to the available EQ-5D-3L value sets (Dolan, 1997; van Hout et al., 2012).

We also included single items that assessed other important aspects of HRQoL. Participants rated their overall upper limb function and appearance on two numeric rating scales (NRS; 0–10, where 10 is best). Hand function in activities of daily living was rated on the following 5-point scale: 1 = I’m able to do all activities myself; 2 = I’m able to do almost all activities myself, and the few things I cannot do are not bothering me, and I never ask for assistance; 3 = I’m able to do most activities myself, but I need assistance for a few activities; 4 = I need assistance for many activities, but I’m able to do some activities myself; and 5 = I need assistance for almost all activities. One question asked if there were any activities they would like to perform better (yes/no). They rated pain at rest and when active on two NRS (0–10, where 0 is no pain and 10 is worst pain).

The participants answered questions about prosthesis wear according to whether they had never worn a prosthesis, currently wore one or had done so previously (Table S1, available online). All were asked to suggest the ideal prosthesis fitting age.

### Statistical analysis

We used the z-test to compare the participants’ mean PROM scores with those of the Norwegian population. These reference scores are given for 10-year intervals with different lower age boundaries and for men and women (Aasheim and Finsen, 2014; Garratt et al., 2022; Garratt and Stavem, 2017). Our sample size was too small to compare participants with the reference values for each 10-year interval, and hence we calculated means and standard deviations (SD) according to the reference population that were as close as possible to our cohort of participants aged 16–52 years; these were age groups 20–49 years for QuickDASH (Aasheim and Finsen, 2014), 18–49 years for EQ-5D-5L (Garratt et al., 2022) and 16–49 years for RAND-36 (Garratt and Stavem, 2017). The EQ-5D-5L, EQ-VAS and RAND-36 are largely reported as means (SD), and so we have also reported the means (SD) for prosthesis wearers and non-wearers. However, because most data were not normally distributed, we have also reported the medians and interquartile ranges (IQRs) and used the non-parametric Mann–Whitney *U* test for independent samples in all comparisons of PROM and single-item scores between prosthesis wearers and non-wearers. We used Fisher’s exact test for nominal data. Post hoc power calculations showed that it would require 14 individuals in each group to detect a 10-point difference in QuickDASH (Gummesson et al., 2003) with a standard deviation of 9.7 points (Dwivedi et al., 2022) at a significance level of α = 0.05 and a power (1-β) of 80%. We set the significance level at α = 0.05.

## Results

**Figure 1.**
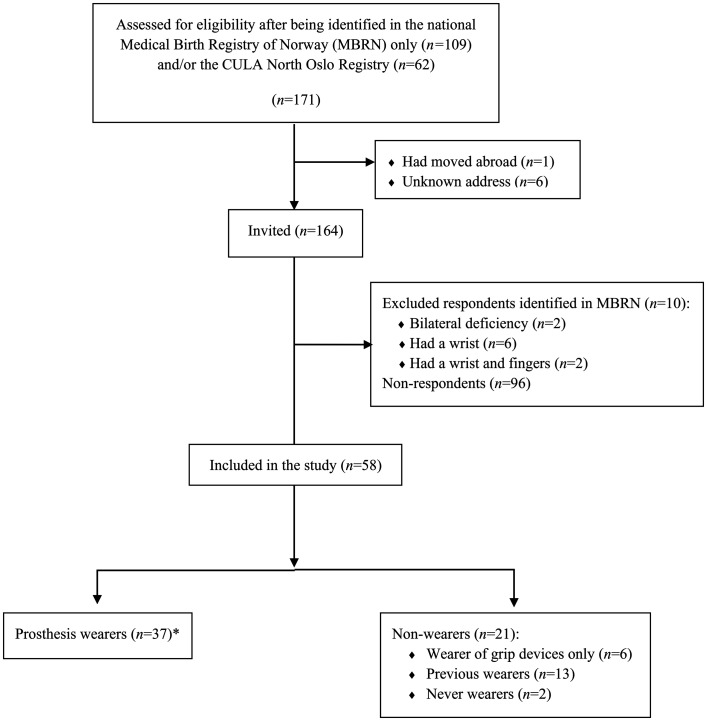
Flow diagram for enrolment of study participants and subgroup division for comparative outcomes analyses. *Prosthesis wearers were defined as persons who currently wore prostheses (daily, weekly or more rarely), as opposed to previous wearers of prostheses and persons who used gripping devices only.

From the two registries, we identified 154 eligible participants (74 women, 80 men). Of them, 58 (38%) responded (Figure 1), of whom 34 (59%) were female, and the median age was 32 years (IQR 23–46). Non-respondents comprised fewer women (42%; *p = *0.047) but were of similar age (median 35 years; IQR 25–44; *p = *0.497). Among the 61 invited individuals identified in the CULA North Oslo Registry, 31 (51%) responded.

The left side was affected in 40 (69%) individuals. The level was at the shoulder in 2 (3%), upper arm in 1 (2%), lower arm in 1 (2%), upper forearm in 48 (83%) and lower forearm in 6 (10%) participants. No other congenital anomalies were reported. Four participants reported having a second-degree relative with the same (*n* = 1) or another (*n* = 3) CULA. Six reported previous operations: resection of a bony prominence (*n* = 2); excision of nubbins (*n* = 2); soft tissue plasty (*n* = 1); and excision of a soft tissue benign tumour (n = 1).

The participants did not have different PROM scores from the Norwegian population (Table 1). Stratified analyses of QuickDASH scores according to the level of deficiency did not indicate that those with a more proximal level of TRDAW had worse upper limb function (Table S2, available online). Responses to single items were skewed towards the better scores. They rated their overall arm function as median 8 (IQR 7–10), and arm appearance as median 7 (IQR 4–10). Of the participants, 40 (69%) reported they could do all activities themselves, 11 (19%) were not bothered by the few things they could not do and never asked for assistance, 6 (10%) needed assistance with a few activities, and one needed assistance for many activities. Of the participants, 29 (50%) reported that there were some activities they would like to perform better. The median NRSs for pain at rest and in activity were 0 (IQR 0–0) and 0 (IQR 0–1), respectively.

**Table 1.** QuickDASH, EQ-5D-5L and RAND-36 scores compared to Norwegian population norms (*n* = 58).

	Sex	*n* ^a^	Study population	Norwegian population^b^	*p*-value^c^
QuickDASH (0–100; 0 best)	F	34	13 (14)	10 (15)	0.24
	M	23	7 (9)	8 (14)	0.73
EQ-5D-5L index (−0.59–1.00; 1.00 best)	F	33	0.871 (0.174)	0.814 (0.214)	0.13
	M	23	0.873 (0.137)	0.818 (0.174)	0.13
EQ-5D VAS (0–100; 100 best)	F	33	73.1 (18.2)	77.7 (17.5)	0.13
	M	23	77.4 (14.3)	78.8 (17.3)	0.70
RAND-36 (0–100; 100 best)					
Physical functioning	F	32	92.97 (9.15)	92.18 (14.08)	0.75
	M	23	95.22 (6.82)	93.69 (12.80)	0.57
Role – physical	F	33	83.33 (32.27)	83.97 (31.57)	0.91
	M	23	81.16 (29.54)	86.93 (28.43)	0.33
Bodily pain	F	33	82.05 (23.82)	80.18 (22.76)	0.64
	M	23	87.39 (16.07)	82.32 (22.01)	0.27
General health	F	33	77.54 (20.01)	77.25 (20.09)	0.93
	M	23	78.04 (15.21)	78.75 (18.79)	0.86
Role – emotional	F	33	83.84 (33.46)	86.87 (28.48)	0.54
	M	23	79.71 (35.87)	89.81 (25.75)	0.06
Vitality	F	33	61.82 (23.68)	58.31 (19.67)	0.31
	M	23	61.96 (18.45)	62.65 (19.25)	0.86
Social functioning	F	33	88.64 (17.78)	87.39 (19.74)	0.72
	M	23	84.78 (20.63)	89.00 (18.76)	0.28
Mental health	F	33	81.70 (16.46)	78.90 (15.08)	0.29
	M	23	78.61 (13.35)	80.15 (14.88)	0.62

Data are expressed as *n* or mean (SD).

a The variation in the number of women (32–34) is because of missing data, and scale scores could not be computed.

b Calculated from the Norwegian population means and standard deviations for the age ranges 20–29, 30–39 and 40–49 for QuickDASH, 18–29, 30–39 and 40–49 for EQ-5D-5L and 15–19, 20–29, 30–39 and 40–49 for RAND-36.

c One-sample two-tailed z-test.

EQ-5D-5L: the 5-Level EuroQol-5-Dimension instrument; F: female; M: male; QuickDASH: the short version of the Disabilities of the Arm, Shoulder and Hand Outcome Measure; RAND-36: RAND 36-Item Short Form Health Survey; VAS: visual analogue scale.

All 58 participants had been offered a prosthesis, and 56 (97%) had tried one or more of the different types (Table 2). A total of 52 reported their starting age: 37 (71%) at 0–1 year; 8 (15%) at 2–5 years; 6 (12%) at 6–16 years; and 1 (2%) at 28 years. The median was 1 year (IQR 0–2.8). Of the 58 participants, 37 (64%; 19 women and 18 men) were current prosthesis wearers, including 25 who reported daily wear of one or more types of prosthesis.

**Table 2.** Number of previous and current prosthesis wearers (*n = *56).

	Passive/cosmetic	Conventional myoelectric	Advanced myoelectric	Hook
Have tried (*n*)	45	51	13	29
Current wearer^a^ (*n*)	25	26	8	7^b^
Daily wearer^c^ (*n*)	12	12	2	3
Wear per day (h)	12 (4–15)	13 (9–16)	5.5 (−)	15 (−)
Weekly wearer (*n*)	8	7	2	0
More occasional wearer (*n*)	5	7	4	3

Values are expressed as n or median (IQR).

^a^Current wearers (*n = *37) were defined as individuals who reported regularly wearing one or several prostheses, further sub-grouped according to daily, weekly or more occasional wear.

^b^One participant did not report how often he wore his hook prosthesis.

^c^Daily wearer (*n = *25) of one or several prostheses.

Of the 58 participants, 44 (76%) had tried gripping devices, and the most common were devices for ski poles (*n* = 37), cycling (*n* = 24), cutlery (*n* = 18), lifting weights (*n* = 13) and car driving (*n* = 5). In addition, they had tried gripping devices for 16 other sports and four musical instruments. Of them, 32 (55%) were current users, of whom six wore the gripping device on a daily basis, with a median of 2 h (IQR 2–3.5) of use per day. Of the 58 participants, 6 (10%; three women and three men) reported currently using gripping devices only, and 15 (26%; 12 women and three men) never used anything.

Most current prosthesis wearers reported a global functional and/or aesthetic benefit (Table 3). Wearers reported using prostheses and gripping devices mostly for regular activities (median NRS 9; IQR: 5–10) and other situations outside the home (median NRS 9.5; IQR 4–10) and less often when at home (median NRS 5; IQR 1–9). Those who used gripping devices only reported infrequent use, both for regular activities (median NRS 1; IQR 0–2), other situations outside the home (median NRS 0; IQR 0–2) and at home (median NRS 0; IQR 0–1).

**Table 3.** Frequency ratings of appearance (*n = *36^a^) and function (*n = *35^a^) in those currently wearing a prosthesis.

	Much better	A little better	No difference	A little worse/less normal	Much worse/less normal
Appearance (*n = *36^a^)	16	8	9	3	0
Function (*n = *35^a^)	18	10	3	3	1

a There were 37 current prostheses wearers, but not all completed these two items.

In total, 13 (22%) participants had stopped wearing prostheses at a median age of 13 years (IQR 12–19). Of them, 10 reported benefits for a time while using it for function (*n* = 7), appearance (*n* = 1) or both (*n* = 2). Three reported no benefit but were happy to have tried.

The distributions of participant age, sex, level of TRDAW and starting age for prosthesis wearing were not significantly different for the prosthesis wearers and non-wearers (Table 4). The two groups had similar scores for QuickDASH, EQ-5D-5L and all but the ‘vitality’ scale of the RAND-36. They rated their overall upper extremity function similarly on NRSs, but the wearers rated their arm appearance higher.

**Table 4.** Background variables and PROM scores for adult wearers and non-wearers of prostheses (*n = *58).

	Wearers (*n = *37)			Non-wearers (*n = *21)^a^			
	*n* ^b^	Median (IQR)	Mean (SD)	*n* ^b^	Median (IQR)	Mean (SD)	*p-*value^c^
Age (years)	37	31 (23–41)		21	35 (26–47)		0.26
Women (*n*)	37	19		21	15		0.17
TRDAW level (*n*)	37			21			0.13
Shoulder and arm		1			3		
Forearm		36			18		
Start age (years)	35	1 (0–2)		17	1 (0–3)		0.50
QuickDASH (0–100; 0 best)	36	7 (0–16)	10 (11)	21	7 (0–19)	12 (14)	0.91
EQ-5D-5L index (−0.59–1.00; 1.00 best)	35	0.879 (0.796–1.00)	0.885 (0.142)	21	0.879 (0.723–1.00)	0.850 (0.184)	0.64
EQ-5D VAS (0–100; 100 best)	36	80 (70–87)	77 (15)	20	75 (54–80)	71 (19)	0.21
RAND-36 (0–100; 100 best)							
Physical functioning	35	95 (95–100)	95 (7)	20	98 (86–100)	93 (11)	0.85
Role – physical	35	100 (75–100)	84 (28)	21	100 (63–100)	80 (36)	0.96
Bodily pain	35	100 (78–100)	85 (21)	21	90 (69–100)	83 (21)	0.52
General health	35	85 (70–90)	80 (15)	21	80 (60–92)	74 (22)	0.36
Role – emotional	35	100 (100–100)	87 (30)	21	100 (33–100)	75 (39)	0.24
Vitality	35	70 (60–80)	68 (15)	21	50 (33–75)	51 (27)	**0.02**
Social functioning	35	100 (88–100)	91 (15)	21	88 (69–100)	81 (23)	0.09
Mental health	35	84 (76–92)	83 (10)	21	84 (62–90)	76 (21)	0.31
Overall arm function (0–10; 10 best)	37	8 (7–10)		21	8 (7–10)		0.84
Arm appearance (0–10; 10 best)	37	8 (6–10)		21	6 (1–9)		**0.03**

Statistically significant *p*-values are shown in bold font.

a Six individuals reported using gripping devices only: daily (*n* = 1); weekly (*n* = 2); and more rarely (*n* = 3). A total of 15 individuals reported never using any prostheses or gripping devices.

b The variation in the number of participants is due to missing data, and scales could not be computed.

c Non-parametric Mann–Whitney *U* test for continuous data, Fisher’s exact test for categorical data.

EQ-5D-5L: the 5-Level EuroQoL-5-Dimension version; PROM: patient-reported outcome measures; QuickDASH: the short version of the Disabilities of the Arm, Shoulder and Hand Outcome Measure; RAND-36: RAND 36-Item Short Form Health Survey; TRDAW: transverse reduction deficiency above the wrist; VAS: visual analogue scale.

In total, 48 (87%) participants had attended multidisciplinary prosthesis clinics where they had met peers:once or twice (*n* = 22) or regularly (*n* = 26). The clinics’ importance for prosthesis wear motivation was rated NRS 5 (IQR 0–7). In total, 38 expressed their opinion on the ideal prosthesis fitting age: 0–2 years or as early as possible (*n* = 28); preschool age (*n* = 7); and when the child expresses a need (*n* = 3).

## Discussion

Nordic infants with TRDAW are routinely fitted with prostheses, although previous research has shown that they confer no benefit in childhood (James et al., 2006), and there is a lack of research on the level of disability in adults. In this cohort study of a homogenous national population of adults with TRDAWs who had been fitted with prostheses free of cost from early childhood, we found that their PROM scores did not differ from those of the general Norwegian population. Neither did PROM scores differ for prosthesis wearers and non-wearers except for ‘vitality’ assessed by the RAND-36 and the NRS-rated appearance. These findings are useful for clinicians when counselling parents of children with TRDAW.

The main limitation of this study was the response rate of 38%. The initial response rate was 44%, but we excluded 10 respondents identified from the MBRN who reported that they had a bilateral deficiency or a more distal deficiency level than TRDAW. Persons with a deficiency level distal to the wrist are not offered functional or cosmetic prostheses in Norway, and hence these respondents were outside the scope of this study. A low response rate might lead to selection bias, which occurred in the sex of the responders, who were more commonly female. The comparisons with the general population reference scores took account of this particular bias because the outcomes are stratified by sex, as women generally report more disability than men (Aasheim and Finsen, 2014; Garratt et al., 2022; Garratt and Stavem, 2017). More detailed information about non-respondents identified only from the MBRN, including the level of deficiency and prosthesis fitting age, was unavailable. This was a limitation of the design: our goal was to include all eligible persons from the whole national population and not just from a selected population or centre where, although such information would have been available, there would be a high risk of selection bias because only those who had sought advice or treatment would be identified. The lack of PROM data from 62% of eligible participants weakens our main findings, and we do not know whether the non-respondents would have scored worse or better than the respondents. Nevertheless, we have regarded the response rate and the cohort size as adequate, and it was sufficiently large to compare outcomes between wearers and non-wearers. It is also possible that the PROMs chosen for this study were not precise enough to assess subtle differences. We did include a range of PROMs, which are the most widely used in orthopaedic research.

Our findings of normal QuickDASH scores and the absence of pain in persons with TRDAWs were similar to those of a recent North American study that recruited from a hospital network population with a 9% response rate, of whom 14% were prostheses wearers (Dwivedi et al., 2022). An earlier Norwegian study (Johansen et al., 2016b, 2018) reported lower SF-36 scores and a high rate of chronic pain in individuals with transverse or longitudinal reduction anomalies but only included those who had actively sought guidance from the National Resource Centre for Rare Disorders, which could have caused selection bias by excluding those who had fewer symptoms or less disability. Their study also had further selection bias for age and sex for respondents compared to the non-respondents. A Dutch study has reported a high degree of musculoskeletal pain and lower scores for RAND-36 scales in a mixed cohort of individuals with reduction deficiencies and acquired amputations (Postema et al., 2016).

We do not regard the normal PROM scores in persons with TRDAWs as indicating that the deficiency is minor, but the findings do highlight differences in perceived disability between persons with congenital anomalies and persons with acquired amputations. Persons with TRDAWs have not lost a body part or previous function in a limb; they have learned from infancy to perform all tasks in life and unlike those with traumatic amputations, they do not experience pain. Another explanation for the good arm function scores could be adaption: persons with TRDAWs might avoid two-handed activities or have fewer expectancies of function than those with two hands.

On the basis of current evidence, we cannot quantify how much wearing a prosthesis affects PROM scores. Nor can we completely recommend or reject prostheses as a therapeutic intervention. The long-term wear of prostheses by more than half of our adult participants can be interpreted as a measure of a perceived functional or aesthetic benefit, or both (Smail et al., 2021), or it might be a result of having been advised to wear a prosthesis since childhood. Activity-specific gripping devices were used by the largest number of individuals in our study, and we could not find previous reports about their use. The difference of 22 points for the RAND-36 ‘vitality’ scale among prosthesis wearers and non-wearers meets a range of recommendations for minimal clinically important differences (MCID) (Bjorner et al., 2007; Wyrwich et al., 2005). It is unclear whether the statistically significantly higher ratings of arm appearance in the group of wearers was clinically relevant, because the MCID for this question is unknown. The benefits from prosthesis wear are possibly multifactorial, with variation between wearers.

Previous studies in children have indicated that those who were fitted with prostheses at an earlier age were more likely to wear them (Davids et al., 2006; Postema et al., 1999; Scotland and Galway, 1983) and early fitting was also recommended by a large proportion of our participants. We could not determine whether an early starting age was associated with prosthesis wear in adulthood because few persons had a high starting age, meaning that a comparison could not be made. Nevertheless, although offering activity-specific gripping devices for daily activities and sports is uncontroversial, we agree with James et al. (2006) that the rationale for prosthesis fitting in infants is questionable. Our findings support advising caregivers that owing to the congenital nature of the condition, children who reject their prostheses will not have worse upper extremity function in adulthood.

## Supplemental Material

sj-pdf-1-jhs-10.1177_17531934231222017 - Supplemental material for Patient-reported function, quality of life and prosthesis wear in adults born with one hand: a national cohort study Supplemental material, sj-pdf-1-jhs-10.1177_17531934231222017 for Patient-reported function, quality of life and prosthesis wear in adults born with one hand: a national cohort study by Ida Neergård Sletten, Kari Klungsøyr, Andrew Garratt and Jarkko Jokihaara: on behalf of the CONTRAST consortium in Journal of Hand Surgery (European Volume)

sj-pdf-2-jhs-10.1177_17531934231222017 - Supplemental material for Patient-reported function, quality of life and prosthesis wear in adults born with one hand: a national cohort study Supplemental material, sj-pdf-2-jhs-10.1177_17531934231222017 for Patient-reported function, quality of life and prosthesis wear in adults born with one hand: a national cohort study by Ida Neergård Sletten, Kari Klungsøyr, Andrew Garratt and Jarkko Jokihaara: on behalf of the CONTRAST consortium in Journal of Hand Surgery (European Volume)
