# Supplementary material for: Patient-reported function, quality of life and prosthesis wear in adults born with one hand: a national cohort study
Source: J Hand Surg Eur Vol. 2023 Dec 21;49(9):1126–33. doi: 10.1177/17531934231222017 (PMC11468110; doi:10.1177/17531934231222017)
Supplement: sj-pdf-1-jhs-10.1177_17531934231222017 - Supplemental material for Patient-reported function, quality of life and prosthesis wear in adults born with one hand: a national cohort study [file sj-pdf-1-jhs-10.1177_17531934231222017.pdf]

Supplementary table 1. Prosthesis wear questionnaire.

Current and previous wearers

Start age (years)________?

Which types of prostheses have you tried?

□ cosmetic
□ hook
□ conventional myoelectric
□ multi-articulating myoelectric
□ grip devices for specific activities:
  □ bicycling
  □ skiing
  □ other sport (*please describe*):_____________
  □ cutlery
  □ driving a car
  □ household (*please describe*):_____________
  □ school (*please describe*):_______________
  □ other (*please describe*):_________________

Have you attended prosthesis clinics in a hospital or orthopedic workshop where you have met other children/adolescents that wore prostheses/grip devices for similar congenital upper limb anomalies?

□ no
□ yes, once or twice
□ yes, regularly

If yes, how important was contact with peers for your motivation for further prosthesis/grip device wear?

no value   very valuable

□ □ □ □ □ □ □ □ □ □ □

Current wearers

Which prostheses/grip devices do you wear now, and how often do you wear them?

1) cosmetic/passive
   □ daily for ___ hours, □ every week, □ less often, □ never
2) hook
   □ daily for ___ hours, □ every week, □ less often, □ never
3) conventional myoelectric
   □ daily for ___ hours, □ every week, □ less often, □ never
4) multi-articulating myoelectric
   □ daily for ___ hours, □ every week, □ less often, □ never
5) grip devices for different tasks
   □ daily for ___ hours, □ every week, □ less often, □ never
   which activities:
     □ bicycling
     □ skiing
     □ other sport (*please describe*):_____________
     □ cutlery
     □ driving a car
     □ household (*please describe*):_____________

□ school (*please describe*):________________
□ work (*please describe*):_________________
□ other (*please describe*):_________________

How often do you wear your prostheses/grip devices:
1) At home, with your family?
never always (except night)
□ □ □ □ □ □ □ □ □ □ □

2) At regular activities outside your home (school, work, hobbies, or with friends)?
never always (except night)
□ □ □ □ □ □ □ □ □ □ □

3) In other places/situations, e.g., with people you don’t know?
never always (except night)
□ □ □ □ □ □ □ □ □ □ □

Which of the following describes best how you *look* with the prosthesis/grip device:
□ much better than without a prosthesis
□ little better than without a prosthesis
□ no difference
□ a little worse and less normal than without a prosthesis
□ much worse and less normal than without a prosthesis

Which of the following describes best how you *function* with the prosthesis/grip device:
□ much better than without a prosthesis
□ little better than without a prosthesis
□ no difference
□ a little worse and less normal than without a prosthesis
□ much worse and less normal than without a prosthesis

Previous wearers

Age when you stopped wearing a prosthesis (years)_____?

Did you have any benefit when you wore prostheses/grip devices?
□ no, I wish I had never had to try it out
□ no, but I am glad that I have tried, so that I know what it is like
□ yes, for a period in life the prosthesis/grip device was beneficial for my function
□ yes, for a period in life the prosthesis/grip device was beneficial for my appearance

Never wearers

Have you been offered prostheses/grip devices?
□ no
□ yes

All

Please suggest the ideal prosthesis fitting age:_____________
(free text, categorized into one of the following: 0-2 years or as early as possible; preschool age; when the child expresses a need).
