# Supplementary material for: Patient-reported function, quality of life and prosthesis wear in adults born with one hand: a national cohort study
Source: J Hand Surg Eur Vol. 2023 Dec 21;49(9):1126–33. doi: 10.1177/17531934231222017 (PMC11468110; doi:10.1177/17531934231222017)
Supplement: sj-pdf-2-jhs-10.1177_17531934231222017 - Supplemental material for Patient-reported function, quality of life and prosthesis wear in adults born with one hand: a national cohort study [file sj-pdf-2-jhs-10.1177_17531934231222017.pdf]

Supplementary Table 2. Upper limb function assessed by QuickDASH stratified by level of deficiency (n=58)

		n	Median (IQR)	Mean (SD)
Shoulder	Females	1	9	9
	Males	1	0	0
Upper arm	Females	1	16	16
	Males	0		
Lower arm	Females	1	7	7
	Males	0		
Upper forearm	Females	28	11 (2 to 22)	15 (15)
	Males	20	5 (0 to 7)	7 (10)
Lower forearm	Females	3	5	5 (5)
	Males	3	7	7 (2)

QuickDASH, the short version of the Disabilities of the Arm, Shoulder and Hand Outcome Measure; n, number of persons; IQR, interquartile range; SD, standard deviation.
